# A Comparative Study of a Small Series of Patients (50 Patients) with Pelvic Varicose Veins Treated with Plugs Alone or Plugs and Polidocanol

**DOI:** 10.3390/jcm12165408

**Published:** 2023-08-20

**Authors:** Miguel Ángel De Gregorio, Masao Yamamoto-Ramos, Arturo Fredes, Carolina Serrano-Casorran, Sergio Sierre, Juan José Ciampi-Dopazo, Santiago Méndez, Jose Maria Abadal, Ignacio Urtiaga, Cristina Bonastre, Jose Rodríguez, Jose Urbano, José Andrés Guirola

**Affiliations:** 1 Minimally Invasive Techniques Research Group—GITMI, University of Zaragoza, Clínica Quirón, 50013 Zaragoza, Spain; 2 Interventional Radiology, Minimally Invasive Techniques Research Group—GITMI, Clínica Quirón, 50013 Zaragoza, Spain; myamamotor@gmail.com; 3 Interventional Radiology, Clinica Quiron Salud Zaragoza, 50006 Zaragoza, Spain; arturofredes@gmail.com; 4 Minimally Invasive Techniques Research Group (GITMI), University of Zaragoza, 50013 Zaragoza, Spain; carolse@unizar.es (C.S.-C.); cbonastr@unizar.es (C.B.); joserodriguez.vet@gmail.com (J.R.); 5 Interventional Radiology, Hospital Universitario Austral, Buenos Aires B1629, Argentina; sergio.sierre@usa.net; 6 EBIR Interventional Radiology, Hospital Virgen de las Nieves, 18014 Granada, Spain; 7 Interventional Radiology, Hospital Universitario Puerta de Hierro, 28222 Madrid, Spain; 8 EBIR Intereventional Radiology, Hospital Universitario Severo Ochoa, 28914 Madrid, Spain; jmabadal@yahoo.es; 9 Vascular Surgery, Minimally Invasive Techniques Research Group (GITMI), 50013 Zaragoza, Spain; martaurtiaga@terra.es; 10 Interventional Radiology, Minimally Invasive Techniques Research Group (GITMI), Hospital Universitario Ramón y Cajal, 28034 Madrid, Spain; jurbano34@gmail.com; 11 Minimally Invasive Techniques Research Group (GITMI), Hospital Clínico Universitario Lozano Blesa, University of Zaragoza, 50009 Zaragoza, Spain; joseandresguirola@gmail.com

**Keywords:** congestion pelvic syndrome, embolization treatment

## Abstract

Level of Evidence: Level 2. Purpose: To compare the safety and efficacy of vascular plug (VP) and vascular plug and polidocanol foam (VPPF) treatments for embolization in pelvic congestion syndrome (PCS). Materials and methods: A comparative, prospective, two-center study enrolled 50 women with PCS from January 2019 to January 2020. The patients were divided into two groups, and embolization was performed with VP (n = 25) and VPPF (n = 25) treatments. The mean age of the patients was 45.6 years ± 6.9. Three clinical parameters were assessed: abdominal pain, dyspareunia, and lower limb pain. The primary outcome (clinical success at 1 yr using a VAS), number of devices, procedure and fluoroscopy times, radiation doses, costs, and complications were compared. The participants were followed-up at 1, 3, 6, and 12 months. Results: At the 1-year follow-up, clinical success did not significantly differ between the two groups (VP vs. VPPF) regarding the improvement of the symptoms analyzed (pelvic pain, dyspareunia, lower extremity pain, and other symptoms (*p* < 0.05)). The mean number of devices per case was 4 ± 1.1 for the VP group and 2 ± 0.31 for the VPPF group (*p* < 0.001). No major complications were recorded in either group. The VPPF group had a significantly longer fluoroscopy time (42.8 min ± 14.2 vs. 25.4 min ± 7) and longer radiation dose (VPPF air kerma 839.4 ± 513 vs. VP air kerma 658.4 mGy ± 355 (all *p* < 0.001)). Conclusions: Embolization for PCS resulted in pain relief in 90% of patients; the use of polidocanol did not demonstrate changes in the clinical outcome. The use of a VP alone was associated with decreased fluoroscopy time and radiation dose.

## 1. Introduction

Pelvic congestion syndrome (PCS), included in a pathology called pelvic venous disorders (PeVDs) in women [1], is a common cause of chronic pelvic pain that occurs in women of reproductive age. The pain may be intermittent or constant with a duration of at least 6 months. PCS can be associated with other symptoms, such as gynecological dysfunction and urinary, intestinal, and pelvic floor disorders [2]. For a long time, PCS has been a misdiagnosed pathology [3]. Chronic pelvic pain (CPC) is often severe enough to cause functional disability and warrant treatment. The prevalence of PCS varies from 6% to 27% worldwide [4].

Pelvic venous congestion may be due to hormonal changes, venous valve insufficiency, or secondary to venous stenosis or obstruction [5,6].

The treatment of pelvic congestion syndrome (PCS) includes hormonal drug therapy and, in some cases, surgical ligation, including hysterectomy. However, endovascular embolization has been shown to be as effective as traditional surgery, while less invasive and better tolerated by patients [7,8].

Various embolizing agents have been used to embolize the gonadal or iliac veins involved in PSC. The purpose of this study is to compare a solid agent (vascular plug alone) (VP) with a solid agent (vascular plug) and a sclerosing agent (polidocanol 2% foam) (VPPF).

## 2. Material and Methods

### 2.1. Study Design

This prospective comparative study was approved by the institutional review board (Reference number CEICA CP-CI PI18/077, 28 March 2018) and was conducted in accordance with the principles of the Declaration of Helsinki. Written informed consent was obtained from all participants for both the procedure and the study. Eligible patients were assigned to two groups without randomization. The patients were consecutively assigned to one of the two groups without considering any differential factor to receive VP or VPPF treatment. All patients were recruited from January 2019 to January 2020. The primary outcome was clinical success subjectively assessed by patients. The secondary outcomes were complications with a need for re-embolization secondary to lack of improvement, recurrence of initial symptoms, or DVTUS revealing venous reflux or venous diameter > 6 mm.

### 2.2. Population Selection

**Figure 1:**
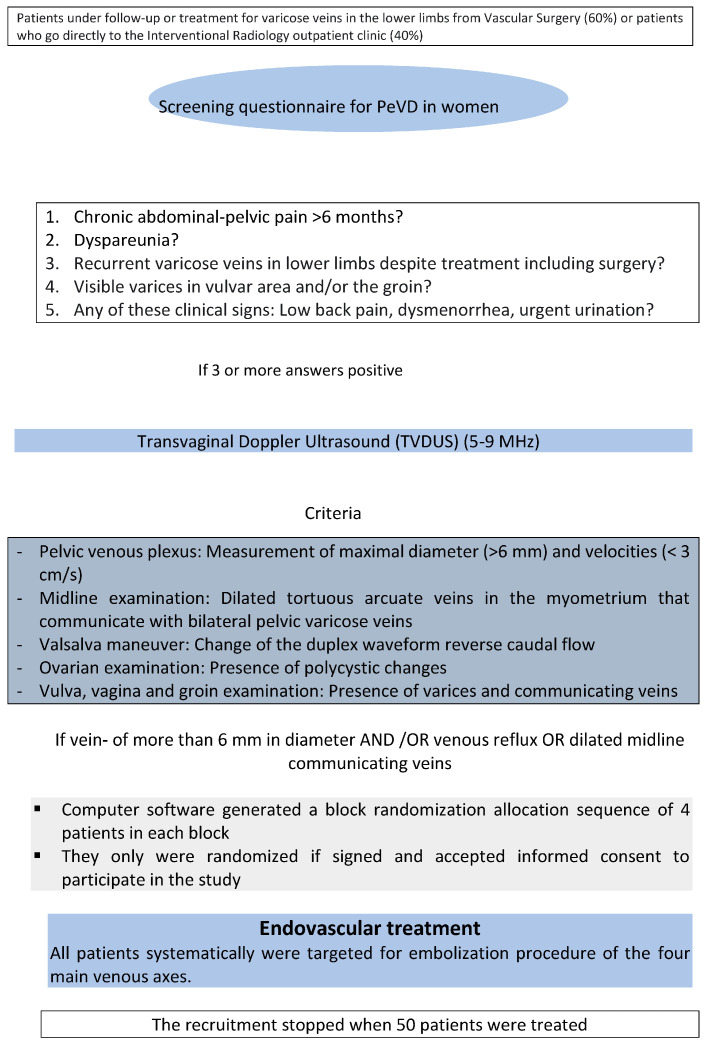
Diagnostic methodology.

**Figure 2:**
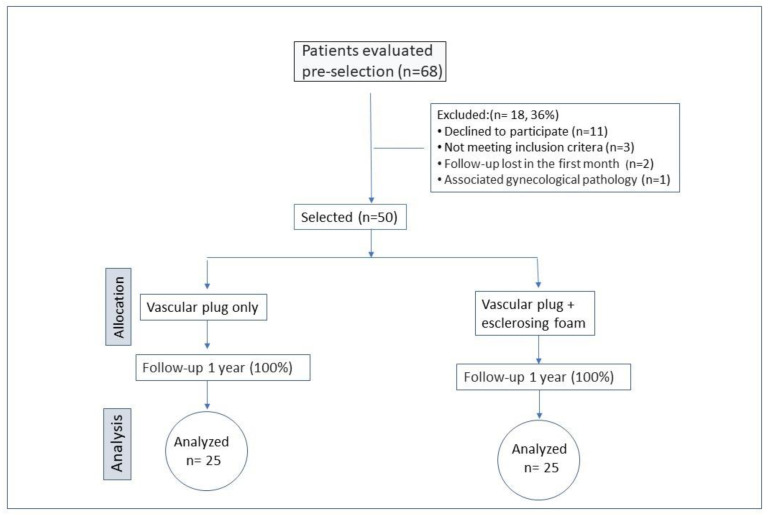
Flow diagram in this study.

This study was conducted at two centers. The patients were referred by various specialists. All women underwent a Transvaginal Doppler Ultrasound (TVDUS) to diagnose pelvic varices and met the eligibility criteria (Figure 1 and Table 1). The diagnosis of PCS involved a combination of symptoms and ultrasound findings (Figure 2). The initial demographic data of the patients in each cohort are presented in Table 2.

### 2.3. Sample Size

The sample size was determined using computer software (G*Power 3.1; Universität Kiel, Kiel, Germany) using prospective data from patients seen in 2019–2020 [9], comparing the mean total procedure time for both methods (44. 5 min ± 6.6 vs. 36.2 min ± 6.6). To detect the differences with an α error of 0.05 and a power of 80% using a two-sided test, 10 patients in each group were considered sufficient. However, for this study, 25 patients were included in each group.

### 2.4. Embolic Agents

A solid medium, such as the Amplatzer Vascular Plug (AVP) (St. Jude Medical, Plymouth, MN, USA), and a liquid medium, a sclerosing agent, were used as embolizing agents. AVP II (Amplatzer) with diameters from 8 to 22 mm and lengths from 6 to 18 mm were used. Depending on its sheath, it requires a 5–8 F caliber catheter [10].

Polidocanol (Kreussler & Co., Wiesbaden, Germany) is a sclerosing and irritating liquid substance formed by the ethoxylation of dodecanol. Injected to treat varicose veins, it causes fibrosis within the varicose veins, occluding the lumen of the vessel and reducing the appearance of varicose veins. A mixture of 2% polidocanol with air and 1 cc of iodinated contrast Iopamiro 300 (Bracco imaging, Milan, Italy) was used. The 2% polidocanol foam was prepared by connecting two 10 mL Luer lock syringes using a three-way stopcock containing 2% polidocanol (Etoxyesclerol, Ferrer Spain, Barselona, Spain) and 8 mL of CO_2_. The contents were mixed until a blend of homogeneous foam was achieved [11].

### 2.5. Technique

Two experienced interventional radiologists, both with >30 years of experience, performed all embolization procedures in both hospitals.

The technique has been described by the authors in previous publications and has not changed substantially for plug embolization (Group VP). In all cases, the four venous axes were closed [8,10].

**Figure 3:**
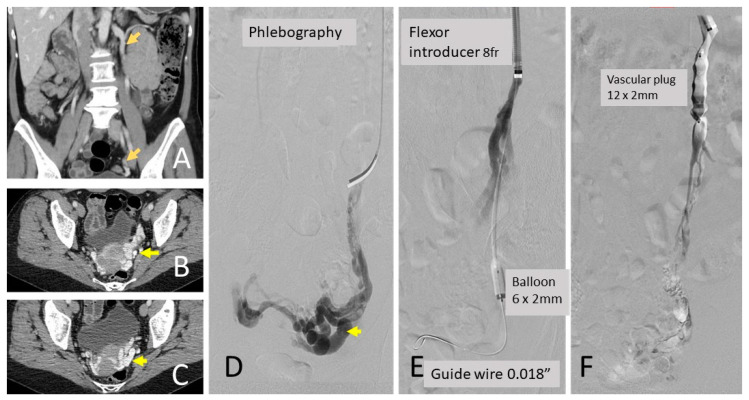
Pelvic varices. CT angiography: (**A**) coronal view shows the large caliber left ovarian vein (arrows), (**B**) axial section with left gonadal varices, (**C**) axial section of peri uterine varices, (**D**) phlebography of left ovarian vein with contralateral reflux, (**E**) phlebography with flexor-type 8Fr catheter, 6 mm × 2 mm balloon occluding the ovarian vein on a 0.018′ microguide. Injection of distal ethoxysclerol; (**F**) ovarian vein occlusion with proximal plug.

In the VPPF group, the existence of direct venous connections to the systemic venous circulation was ruled out. When these were found, the connection and flow were reduced by releasing a VP of the appropriate caliber. On a few occasions, it was required to close the connection with an occlusion balloon. Coaxially to the MPA 5F catheter, a 2.7 F 130 cm Progreat microcatheter (Terumo, Tokyo, Japan) was introduced, and a foam of 2 mL of 2% polidocanol was mixed with 8 mL of air and 1 mL of iodinated contrast. This mixture was injected slowly until the vessel was obstructed, verifying that there was no migration of the foam towards the systemic venous circulation. Occlusion (lOV, ROV, LHV, and RHV) was confirmed through venography performed after embolization (Figure 3). During the intervention, all the data were collected.

## 3. Outcomes

Clinical success was defined as the relief of symptoms experienced before the procedure, including abdominal pain, dyspareunia, lower limb pain, dysmenorrhea, and urinary urgency (assessed by direct questioning before the procedure and after 1, 3, 6, and 12 months). The subjective abdominal pain, dyspareunia, and lower limb pain was assessed with a VAS scored from 0 (no pain or symptoms) to 10 (worst pain or symptoms possible). Two categories were created: relief of symptoms (VAS score decreased by 4 points) and no improvement or worsening (new symptoms, increase or no change in VAS score, or improvement in VAS score < 3 points) [10]. The secondary endpoints were a technical success (feasibility of embolization of the four targeted veins): number of devices used, total procedure time from venous puncture to venous compression, total fluoroscopy time, radiation dose (DAP and total air kerma recorded using the fluoroscopy equipment), overall complications, and need for re-embolization (scheduled during the subsequent 12 months).

## 4. Follow-Up

The patients in both groups had clinical follow-up at 1, 3, 6, and 12 months after the embolization procedure. The related symptoms were recorded, and subjective pain was assessed by the patients at each follow-up visit. When no improvement was observed, initial symptoms recurred, or DVT showed venous reflux or persistent venous diameter > 6 mm, re-embolization was scheduled.

To assess quality of life, a telephone survey was conducted (at 12 months). The patients were asked about abdominal pain, sexual intercourse, and pain in the lower limbs. Each question was scored from 0 to 10 according to the following distribution: almost always = 0, often = 1–3, sometimes = 4–6, occasionally 7–9, and never = 10. At the end of this study, a survey was conducted, including regarding the quality of the telephone service, with patients scoring their satisfaction with the procedure: the treatment received and the possibility of recommending it to other patients from 0 to 10.

## 5. Statistical Analysis

By applying the Kolmogorov–Smirnoff test, normality was evaluated. The mean, standard deviation, and range were used to express quantitative variables. Total events and percentages were used to convey qualitative data. The parametric Student t test and the nonparametric Mann–Whitney U test were used to assess continuous data. For categorical variables, the Thec2 test and Fisher exact test were employed. A scatterplot was used to represent time data, and a line chart was used to show how the VAS score changed over time (1, 3, 6, and 12 months). *p* < 0.05 was regarded as significant in all two-sided tests of significance. Using IBM SPSS Version 21.0 (IBM Corp, Armonk, New York, NY, USA), data were processed and examined.

### Results

Fifty patients were randomly assigned to the two groups. Except for those excluded in the first month, no patients were lost at 3, 6, or 12 months of follow-up, and all 50 patients were analyzed. See Figure 2.

## 6. Treatment

In both groups, the technical success was 100%. In all cases, it was possible to treat the four venous axes (ovarian and hypogastric). Tin toto achieve total occlusion of the four veins in the VP group, a mean of 4.2 plugs was used. The sclerosing substance was never used in this group. In the VPPF group, a mean of 2.2 plugs were used, and all four venous axes were sclerosed in 21 (84%) patients. Sclerosis in the hypogastric veins was ruled out in four patients because they had a very wide communication with the external iliac system (*p* < 0.0001). The total procedure time, total fluoroscopy time, and radiation dose were significantly lower in the VP group compared to the VPPF group (Table 3).

**Figure 4:**
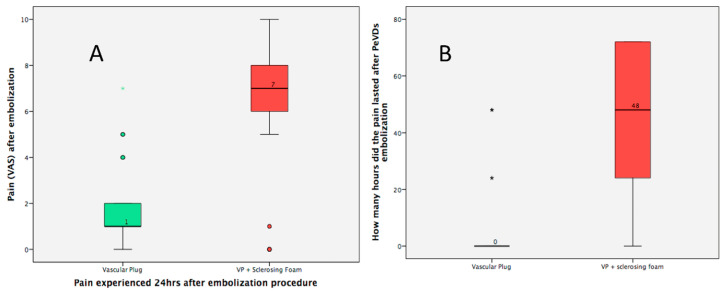
Diagram showing: (**A**) intensity of pain after embolization and (**B**) duration of pain in hours.

There were no major complications in the VP group, except for a small hematoma in the jugular access that did not require additional measures, and four patients reported mild pelvic pain of short duration that resolved with oral analgesia. In the VPPF group, a neck hematoma was confirmed that did not require additional measures, and 20 patients (80%) presented pelvic pain with a mean intensity VAS of 6.4 ± 3.1 (range 0–10) with a duration of 40 min ±27 h (range 0–72 h) that required treatment with Metamizole and/or Tramadol. Higher VAS values and longer duration of pain were observed in the VPPF group (Figure 4A,B).

## 7. Clinical Outcomes

Clinical success was achieved at 1-year follow-up in all 50 patients. Table 4 shows the results at 12 months of follow-up in comparison with symptoms that existed before treatment.

At 1-year follow-up, clinical success did not differ significantly between the two groups (VP vs. VPPF) in terms of improvement in the symptoms analyzed (pelvic pain, dyspareunia, lower extremity pain, and other symptoms).

In both groups, the relief of symptoms was found in the VAS for abdominal pain and dyspareunia, pain in the lower limbs, and other symptoms (there was a decrease of >4 points with respect to the pre-treatment score) in 88%, 96%, and 48% of patients, respectively. The VAS for lower limb pain and other symptoms showed no worsening but no change in 52% of patients.

Two patients’ symptoms in each group did not improve (score <3 points) in a corresponding way when the four values (abdominal pain, dyspareunia, lower extremity pain, and other symptoms) were analyzed compared to when only abdominal pain was analyzed. Of the patients whose symptoms did not improve, two of them, one in each group, underwent a second embolization at 5 and 10 months, respectively; despite this, the symptoms did not improve substantially.

The satisfaction score, personal treatment, and recommendation interest at the end of this study, according to the quality questionnaire carried out by telephone, can be seen in Table 5. No significant differences were found between the two groups (p 0.146). See Table 5.

## 8. Discussion

It has been described that embolization produces total or partial improvement in symptoms in between 60 and 100% of patients diagnosed with pelvic venous disorders (PeVDs) [12]. Despite multiple treatments having been proposed [13,14], transcatheter embolization is currently the treatment of choice in the management of pelvic congestion syndrome due to varicose veins [12,15]. There is not very strong clinical evidence (grade 2); however, there are many data in long series with a follow-up of more than 4–5 years that support the efficacy and benefits of this treatment [7,8,12,16,17]. Various materials have been proposed to perform embolization, including solid agents (coils and plugs) [8,10] used alone or in combination with liquid agents (sclerosing substances, cyanoacrylates) [11,17,18,19] and even ethylene-vinyl alcohol (EVOH) [20]. Even though this range of agents has been used in different series, no great differences have been observed between one agent and another [3].

Although the guidelines, the consensus, and meta-analyses [15,17,21,22] invite randomized studies with different embolization agents or various techniques, there are no randomized studies, except for the one carried out by Guirola A et al. in 2018 [10] in which embolization with coils and plugs is compared. The present randomized study has used vascular plugs in both arms, and in one of the arms, whenever possible, a sclerosing agent was used (polidocanol at 2%). Similarly to the study of Guirola et al. [10], the present study did not demonstrate better clinical results in any of the arms but did show a significant reduction in the number of VPs in the group in which VPPF was used (4–2). However, the VPPF group showed a longer fluoroscopy time (*p* > 0001) and therefore a higher air kerma and DAP (Table 3). Also, 20 patients (80%) in the VPPF group presented pelvic pain with a mean intensity VAS of 6.4 ± 3.1 (range 0–10) with a duration of 40 ± 27 h (range 0–72) hours that required Metamizole and/or Tramadol treatment.

In this study, both treatments had high technical and clinical success rates, as shown in other noncomparative studies [3,11,21,22,23,24,25,26]. There were no differences in the efficacy of the devices. Complete occlusion confirmed with venography was achieved with both VPs and VPPF.

It has been shown that the use of a plug reduces the time of the procedure since it is a fast and effective technique, as well as being simple [10]. The main limitation of sclerotherapy is the need to control the escape of the sclerosing agent into the systemic circulation using occlusion coils, plugs, or balloons that reduce the flow and prevent the migration of the sclerosant. Some authors [3,11] routinely use distal microcatheter embolization with good results and few complications. Another limitation of liquid agents (sclerosants, glues, EVOH) is postprocedural abdominal or pelvic pain that requires potent analgesia, including opioids, that may be required for several days [3,11,27]. In our study, we did not use a microcatheter on a regular basis; however, we did not have any serious complications.

Migration is an important complication [21]. In our study, no migration was observed.

Among the main limitations of this study are the sample size and the significant differences between age groups. However, these differences had no clinical impact (VAS severe pain scores of 7 to 9). Another limitation is that clinical success, referring to abdominal pain and pain and discomfort in the lower limbs, is difficult to measure objectively. A subjective scale, such as the VAS, can be influenced by other concomitant disorders and environmental or psychological conditions [27].

## 9. Complications

While complications arising from venous embolization are infrequent, certain complications can bear clinical significance. Both minor and severe complications are plausible and may manifest during the surgical procedure, shortly thereafter, or during the postoperative follow-up period. This can include vein thrombosis, entry-point hemorrhage, changes in heart rate due to right heart passage, inadvertent release of embolizing agents into unintended veins, material migration, venous rupture, and iodinated contrast adverse reactions.

Of the studies encompassed within the systematic review by Daniels et al. [28], six studies reported no complications. Conversely, 16 studies, involving a total of 938 participants, disclosed that a mere 0.9% of patients presented vein perforation with contrast extravasation. This metanalysis showed that transient pain occurred in between 8% and 100% of patients when sclerotherapy was employed. Notably, this investigation documented 1.1% (eleven cases) of coil migrations, with eight instances occurring in pulmonary vasculature, two within the renal vein, and one within the femoral vein. The retrieval of these migrated coils was achieved utilizing a snare with the endovascular approach.

In a survey conducted by Leal Monedero [24] in 2006, involving a cohort of 239 women, it was revealed that 54% of patients experienced brief gluteal or lumbar discomfort subsequent to embolization. Moreover, 26% reported general pain, 9% displayed elevated body temperatures below 38 degrees Celsius, and 21 patients exhibited local phlebitis at the access site.

Given the paucity of research pertaining to its investigation, the precise impact of vein embolization on fertility remains unknown, although instances of pregnancies after embolization are well documented [17,28,29].

The main long-term complications include migration to the lung, thrombosis of the access vein, foreign body reactions, and compression-induced neuralgia. Conversely, immediate complications primarily entail pain, abdominal discomfort, and the unintended migration of embolizing agents.

## 10. Conclusions

Henceforth, whether employing venous plugging (VP) or venous plugging plus polidocanol foam (VPPF), the practice of venous embolization as a therapeutic intervention for pelvic congestion syndrome (PCS) demonstrates equivalent levels of safety and efficacy. Both modalities yield a substantial alleviation of pelvic symptoms associated with PCS. Clinically, the outcomes of both procedures reveal a comparable attainment of varicose vein occlusion.

In a direct comparison of the VPPF and VP techniques, it is evident that the VPPF procedure requires a longer duration and exposure to radiation. Furthermore, the occurrence of postprocedural abdominal discomfort is notably more frequent in cases of VPPF as opposed to VP, consequently requiring analgesic intervention as a usual measure.

## Figures and Tables

**Table 1:** Inclusion and exclusion criteria.

Inclusion Criteria	Exclusion Criteria
▪ Signed informed consent	▪ Diagnosed gynecological or pelvic pathology: endometriosis
▪ Age > 18 years	▪ Pelvic inflammatory disease, postoperative adhesions, adenomyosis, or leiomyoma
▪ Chronic abdominal or pelvic pain for >6 months	▪ Glomerular filtration rate < 60 mL/min
▪ Pelvic venous diameter > 6 mm by TVDUS	▪ History of contrast agent reaction (relative)
▪ Presence of venous reflux or communicating veins by TVDUS	▪ Patients not able to be followed-up for at least 1 year

**Table 2:** Patient demographic data: ROV = right ovarian vein, LOV = left ovarian vein, RHV = right hypogastric vein, LHV = left hypogastric vein, IVC = inferior vena cava, BVBP = basal venous blood pressure, VVNP = Valsalva venous blood pressure, BMI = body mass index, PF = polidocanol foam, VAS = visual analog scale; * Student *t* test. † Mann–Whitney U test. ‡ Fisher exact test. § c2 test.

Characteristic	Plug (n = 25)	Plug + PF (n = 25)	*p*
Age and mean ± SD	47.1 ± 6.6 (32–58) y	43 ± 6.7(31–60) y	0.012 *
Living children, median and range	1.8 ± 0.6 (1–3)	1.8 ± 1.2(0–4) y	0.967 †
BMI, mean ± SD	26.1 ± 2.0	27 ± 2.5	0.750 *
Symptoms			
Pre-treatment VAS score	7.8 ± 0.8 (6–9)	7.9 ± 1.3 (6–10)	0.839 †
Dyspareunia	6.1 ± 2.8 (0–9)	7.0 ± 3.4 (0–10)	0.047 ‡
Lower limb pain mean ± SD, range	3.3 ± 2.2 (0–6)	4.6 ± 2.2 (0–8)	0.021 §
Associated hemorrhoids	11 (44%)	6 (24%)	0.227 §
Other symptoms mean ± SD, range Dysmenorrhea, urinary urgency	1.1 ± 2.1 (1–2)	1.08 ± 2 (1–3)	0.023 §
Nutcracker Phenomenon	1/25 (4%)	1/25 (4%)	1 §
History			
Ovarian Cyst disease	11 (44%)	11(44%)	0.395 §
Vaginal varicosities	12 (48%)	16 (64%	0.572 §
Vulvar varicosities	10 (40%)	15 (60%)	0.413 §
Lower limb varices	20 85%	20 (85%)	1.00 §
Limb varices surgery	4.16%	5 (20%)	0.467 §
Transvaginal Doppler Ultrasound (TVDUS)			
Maximum right pelvic venous caliber, median and range (mm)	5.6 ± 1.1 (5–9)	5.6 ± 1.4 (4–8)	1.0 *
Maximum left pelvic venous caliber, median and range (mm)	7.2 ± 0.9 (5–9)	7 ± 1.5 (5–9)	0.775 *
Ovarian vein reflux	25 (100%)	25 (100%)	1.0 *
Waveform change in Valsalva maneuver	24 (96%)	21 (84%)	0.192 §
Varicocele	11.44%	17 (68%)	0.395 §

**Table 3:** Intraoperative data: DAP= dose area product; VP = vascular plug; VPPF = vascular plug and polydocanol foam; * Mann–Witney U test; † Student *t* test.

Intraoperative Data	VP = 25	VPPF = 25	*p*
Vascular plugs (median, range)	4 (4–6)	2 (2–4)	0.004 †
Polydocanol foam 2% (median, range)	0	4.5 mL 4–6 mL	0.000
Cost of embolization devices, €, median; range	4401.5, 3523–5280	2641; 1760–3523 *	0.005
Total procedure time, min, mean ± SD; range	31.7 ± 7.1; 17–41	42.8 ± 14.2; 29–90	0.001 †
Fluoroscopy time, min, mean ± SD; range	25.4 ± 7; 14–38	34.2 ± 13.7; 21–78	0.004 †
DAP, mGy cm^2^, median; range	235,413; 115,642–612,503	222,816; 151,871–612,586	0.014
Total air kerma mGy, median; range	658.4; 112–1458	839; 356–2937.0	000

**Table 4:** Assessment of symptoms before and after treatment at 1 year. VP = vascular plug; VPPF = vascular plug and polidocanol foam.

Group	Pre-Embolization (VAS)				Post-Embolization (VAS) 12 Months			
	Abdominal Pain	Dyspareunia	Lower Limb Pain	Other symptoms	Abdominal Pain	Dyspareunia	Lower Limb Pain	Other Symptoms
VP	7.8 ± 0.8 range (6–9)	6.1 ± 2.8 range (0–9)	3.3 ± 2.2 range (0–6)	5.40 ± 2.6 range (0–8)	1.3 ± 0.8 range (0–6)	1.0 ± 0.9 range (0–6)	1.6 ± 0.9 range (0–5)	2.8 ± 3.3 range (0–8)
VPPF	7.9 ± 1.3 range (6–10)	7.0 ± 3.4 range (0–10)	4.6 ± 2.2 range (0–8)	4.12± 3.1 range (0–8)	1.3 ± 1.1 range (0–6)	1.0 ± 0.9 range (0–5)	1.6 ± 0.8 range (0–3)	2.68 ± 3.2 range (0–8)
*p*	>0.05	>0.05	>0.05	>0.05	>0.05	>0.05	>0.05	>0.05

*p* > 0.05, no significance.

**Table 5:** Phone quality survey results. Phone quality questionnaire: degree of satisfaction, personal treatment, and possibility of recommendation.

Group	Degree of Satisfaction with the Procedure	Personal Treatment during the Procedure	Would She Recommend This Treatment to Other Patients (Friends, Relatives...)
VP	7.5 ± 0.9, range 5–9	7.4 ± 1.1, range 4–9	6.7 ± 1.4, range 4–9
VPPF	8 ± 0.7, range 6–9	8.1 ± 0.9, range 6–9	7.5 ± 1.4, range 3–9
TOTAL	7.7 ± 0.8 (range 5–9)	7.7 ± 1.1, range 4–9	7.1± 1.4, range 4–9

## Data Availability

The data presented in this study are available on request from the corresponding author. The data are not publicly available due to privacy restrictions.

## Abbreviations

PCS	Pelvic congestion syndrome
PeVDs	Pelvic venous disorders
USTV	Ultrasound Transvaginal
USDTV	Ultrasound Transvaginal
CPP	Chronic pelvic pain
VP	Vascular plug
VPPF	Vascular plug and polidocanol foam
ROV	Right ovarian vein
LOV	Left ovarian vein
RHV	Right hypogastric vein
LHV	Left hypogastric vein
TVDUS	Transvaginal Doppler Ultrasound
